# Effects of Infection by *Trypanosoma cruzi* and *Trypanosoma rangeli* on the Reproductive Performance of the Vector *Rhodnius prolixus*

**DOI:** 10.1371/journal.pone.0105255

**Published:** 2014-08-19

**Authors:** Maria Raquel Fellet, Marcelo Gustavo Lorenzo, Simon Luke Elliot, David Carrasco, Alessandra Aparecida Guarneri

**Affiliations:** 1 Vector Behaviour and Pathogen Interaction Group, Centro de Pesquisas René Rachou, Fundação Oswaldo Cruz, Belo Horizonte, Minas Gerais, Brazil; 2 Department of Entomology, Universidade Federal de Viçosa, Viçosa, Minas Gerais, Brazil; 3 Unit of Chemical Ecology, Department of Plant Protection Biology, Swedish University of Agricultural Sciences, Alnarp, Sweden; Tulane University, United States of America

## Abstract

The insect *Rhodnius prolixus* is responsible for the transmission of *Trypanosoma cruzi*, which is the etiological agent of Chagas disease in areas of Central and South America. Besides this, it can be infected by other trypanosomes such as *Trypanosoma rangeli*. The effects of these parasites on vectors are poorly understood and are often controversial so here we focussed on possible negative effects of these parasites on the reproductive performance of *R. prolixus*, specifically comparing infected and uninfected couples. While *T. cruzi* infection did not delay pre-oviposition time of infected couples at either temperature tested (25 and 30°C) it did, at 25°C, increase the e-value in the second reproductive cycle, as well as hatching rates. Meanwhile, at 30°C, *T. cruzi* infection decreased the e-value of insects during the first cycle and also the fertility of older insects. When couples were instead infected with *T. rangeli*, pre-oviposition time was delayed, while reductions in the e-value and hatching rate were observed in the second and third cycles. We conclude that both *T. cruzi* and *T. rangeli* can impair reproductive performance of *R. prolixus*, although for *T. cruzi*, this is dependent on rearing temperature and insect age. We discuss these reproductive costs in terms of potential consequences on triatomine behavior and survival.

## Introduction

Chagas disease is a severe infection whose etiological agent is the protozoan *Trypanosoma cruzi* (Chagas, 1909). This parasite is transmitted to humans by insect vectors belonging to the subfamily Triatominae and the main tool to combat transmission of this disease is vector control, based on extensive use of residual insecticides [1], [2]. *Rhodnius prolixus* (Hemiptera: Reduviidae) Stål, 1859 is considered the primary vector of Chagas disease in Venezuela and Colombia but has also been responsible for disease transmission in much of Central America [3]–[5]. Beyond transmitting *T. cruzi*, this triatomine can also be infected by *Trypanosoma rangeli* Tejera, 1920, a protozoan that does not cause disease to humans. This parasite does, however, share surface antigens with *T. cruzi* [6], potentially leading to serological cross-reactivity and so misleading diagnosis of Chagas disease [7], [8].

These two parasites have distinct tropisms in the insect - *T. cruzi* multiplies exclusively in the digestive tract while *T. rangeli* initially infects insects through a similar process but is eventually able to cross the gut epithelium and invade the hemocoel and salivary glands [9]. To grow and multiply, parasites must obtain nutritional resources from their insect hosts. As a consequence, hosts may reorganize metabolic pathways and invest some of their energetic reserves in parasite elimination [10], [11], [ for review see 12]. Therefore, parasite infections have the potential to affect their insect hosts in various ways, directly or indirectly, with possible effects on fitness [13]–[16].

Conventionally, *T. cruzi* has been considered to be non-pathogenic to its insect hosts [17]–[20]: it normally does not increase mortality rates under optimal conditions (e.g. with unlimited food) [18], injure intestinal tissues [19] or even affect populations of symbionts [20]. There have, though, been some reports of alterations in developmental parameters [21], [22] and reproduction [23] of infected insects, depending on the parasite strain and the conditions to which insects were submitted, while some authors also suggest that the parasite may influence wing morphology [24] and dispersion patterns [25].

In contrast to *T. cruzi*, *T. rangeli* is known to be pathogenic to triatomines [9], [26]–[32]: generally, infected insects have difficulties feeding [33] and have increased mortality [32], [34] while those that survive have longer development times [15] and are damaged during ecdysis, leading to altered morphology [25], [35]. In addition, it has been shown that *T. rangeli* can reduce symbiont populations [26], [28] producing several negative effects on insect development [20].

Most studies to date have focused on effects of *T. cruzi* and *T. rangeli* on insect development, longevity and survival. In addition, there are still several controversies regarding the effect of these parasites on the fitness of triatomines. At least some of the differences in the results might be explained by the use of different methodologies and strains and evaluation of the infection of indistinct species of insects and for short periods. In the case of *T. rangeli*, to our knowledge, there are no studies to date showing the effects of the parasite on triatomine reproductive parameters.

We therefore infected *R. prolixus* with *T. cruzi* or *T. rangeli* and examined the effects of these parasites on reproductive aspects of adults. We evaluated pre-oviposion time (time to lay the first egg), the e-value (the insects' capacity to convert ingested blood into eggs) and egg viability. Since in the field triatomines can remain infected for long periods, the effect of infection on aspects of fitness was evaluated over several reproductive cycles. Our results indicate that these parasites affect, to differing degrees, most reproductive aspects we investigated.

## Materials and Methods

### Triatomines

All *R. prolixus* used in assays were obtained from a laboratory colony. This colony is derived from insects collected in Honduras around 1990. The colony was maintained by the Vector Behaviour and Pathogen Interaction Group in Centro de Pesquisas René Rachou, FIOCRUZ, MG, Brazil. Triatomines were reared, until infection, at 25±1°C, 60±10% RH and kept under a natural illumination cycle.

### Ethics Statement

Before and after infection, in all instars, insects were fed on Swiss mice under Thiopental 2.5% anesthesia to minimize animal suffering. To infect the insects we used citrated rabbit blood obtained from Cecal (Centro de Criação de Animais de Laboratório) – Fundação Oswaldo Cruz (FIOCRUZ), RJ, Brazil. All experiments using live animals were performed in accordance to FIOCRUZ guidelines on animal experimentation and were approved by the Ethics Committee on Animal Experimentation (CEUA/FIOCRUZ) under the approved protocol number L-058/08. The protocol is from CONCEA/MCT (http://www.cobea.org.br/), which is associated with the American Association for Animal Science (AAAS), the Federation of European Laboratory Animal Science Associations (FELASA), the International Council for Animal Science (ICLAS) and the Association for Assessment and Accreditation of Laboratory Animal Care International (AAALAC).

### Parasites

*Trypanosoma cruzi* (CL strain) and *T. rangeli* (CHOACHI strain) epimastigotes were used to infect insects. These parasites were first isolated from naturally infected *T. infestans* [36] and *R. prolixus* [37], respectively. Most studies of the interaction between *T. cruzi* and *R. prolixus* use the DM28 strain (11 studies from a total of 13 found), which was originally isolated from the opossum *Didelphis marsupialis* [38]. The CL strain was chosen for the present study since it was isolated from a triatomine bug (rather than a vertebrate), albeit a distinct genus. The strains were cultured by two weekly passages in liver-infusion tryptose (LIT) medium supplemented with 15% fetal bovine serum, 100 mg/ml streptomycin and 100 units/ml penicillin [15].

### Infection by trypanosomes

Infection by *T. cruzi* was induced in second instar nymphs (8±2 days old) to ensure a chronic infection in the adult phase. Inactivated (56°C, 30 min) citrated rabbit blood containing 5×10^6^ parasites/ml was offered to triatomines at 37°C through an artificial feeder. Control group insects received parasite-free blood under the same conditions. The blood was stirred every 20 minutes to ensure homogeneity of parasite distribution during *ad libitum* bug feeding. Subsequently, insects that had fed to repletion were selected for the experiments. After this procedure, insects were split in two groups: one that was maintained in a chamber at 25°C during nymphal development and also after imaginal moult, hereafter called 25–25°C, and another that was maintained in a chamber at 30°C during nymphal development and transferred to 25°C after imaginal moult, hereafter called 30–25°C. Insects were maintained in 12∶12/L:D. The intention in rearing a group at 30°C was to induce a higher parasite load in these insects. Briefly, the previous experiments were performed in our laboratory and have proven that the growth rate of *T. cruzi* cultures is linearly and positively dependent on temperature over the range of 20–30°C [unpublished data]. Infection status was confirmed for all insects used in the experiments through microscopic examination of a drop of urine after blood feeding at the 3^rd^ instar. Insects were fed 8–10 days after each molt in order to ensure continuity of infection [39], [40].

Similarly, infection by *T. rangeli* was achieved by feeding 3^rd^ instar nymphs (8±2 days old) through an artificial feeder on inactivated (56°C, 30 min) citrated rabbit blood at 37°C, containing 1×10^5^ parasites/ml. Nymphs belonging to the control group received parasite-free blood offered in identical conditions. To ensure that all insects contained parasites in hemolymph, seven days after moulting to 4^th^ instar, nymphs were inoculated in the side of the thorax with 1 µl of PBS (0.15 M NaCl 0.01 M sodium phosphate, pH 7.4) containing 2.5×10^4^ parasites/ml. A 50 µl syringe (Hamilton, needle 13×3.3; ½″) connected to a dispenser (model 705, Hamilton Company, USA) was used to inoculate the parasites. Nymphs belonging to the control group were inoculated with the same volume of PBS. Twenty-four hours post-inoculum, insects were allowed to feed *ad libitum* on anesthetized Swiss mice.

Hemolymph examination was performed under the microscope to confirm the infection of all experimental insects. Control insects had a drop of hemolymph sampled in the same manner to ensure equivalent manipulation of the insects studied. Both groups of insects were always maintained in a chamber at 25°C and 12∶12L/D and are hereafter called *T. rangeli* 25–25°C. No experiment was performed with insects infected with *T. rangeli* and exposed to 30°C due to the fact that previous experiments performed in our laboratory have shown that the culture growth rate of these parasites presents a peak at intermediate temperatures, i.e., 25°C, over the range 20–30°C.

For all experiments, both with *T. cruzi* and *T. rangeli* infected insects, we promoted early infections to generate a chronic pathological profile on the insects. We suggest that evaluating reproductive performance on adults infected as younger instars would allow showing effects accumulated throughout their lifespan. Infections by *T. cruzi* or *T. rangeli* were not conducted at the same instar since our main purpose was not to compare the effects of different parasites. As *T. rangeli* infected insects needed the injection of parasites into their hemocel after being orally infected, this had to be performed at a larger instar to avoid mortality due to this damaging procedure. In the light of existing knowledge on triatomine-trypanosome interactions, there is no evidence of parasite-induced virulence being dependant on the instar infected.

### Bioassays

Fifteen-day-old virgin adults (previously individualized as 5^th^ instar nymphs) were individually weighed before and immediately after feeding on anesthetized mice for 30 minutes or until the total distension of abdomen. Afterwards, insects were sorted into breeding pairs which were maintained for 21 days in separate plastic containers (5.5×8.0 cm). Inside each container a circular piece of filter paper and a strip of cardboard were added as substrate and climbing structure. The container was sealed with cloth. The feeding procedure was repeated with the same insects every 21 days for three times. Intervals between feeding were defined as reproductive cycles. The insects remained inside the containers until the end of the experiments or female death. Dead males were not replaced and females were kept alone until the end of the experiments. Only data generated from insects that fed in each cycle were considered for the experiment. After the first imaginal meal, bugs that did not engorge were excluded and the final number of experimental pairs established for each group (trying to keep figures as even as possible). It is relevant to mention that data obtained with a small proportion of pairs had to be excluded from the datasets of the 2^nd^ and 3^rd^ oviposition cycles (both for control and infected groups) as they had either not fed or died. The numbers of pairs for which data were obtained in the 2^nd^ and 3^rd^ cycles is given in figures.

Several experimental series were conducted to examine the effect of infection on the fecundity and fertility of *R. prolixus*.

Four treatments were used to study the effect of infection by *T. cruzi*:

- uninfected pair (25–25°C) (n = 15 pairs);
- infected pair (25–25°C) (n = 19 pairs);
- uninfected pair (30–25°C) (n = 20 pairs);
- infected pair (30–25°C) (n = 20 pairs).

Two treatments were developed to study the effect of infection by *T. rangeli*:

- uninfected pair (25–25°C) (n = 20 pairs);
- infected pair (25–25°C) (n = 20 pairs).

The eggs produced by each pair were collected daily and transferred to a plastic microplate (24 wells). The following parameters were recorded for each pair: a) pre-oviposition time (number of days spent from pairing to laying the first egg); b) e-value for each reproductive cycle [41], [42]; c) egg hatching rate for each reproductive cycle. The *e-value* is a variable that indicates the capacity of bugs to convert the ingested blood into egg production, taking in consideration the initial weight of the female in each cycle. This variable was calculated using the formula: 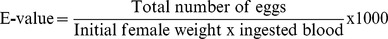



### Statistical Analysis

All the statistical analyses were done in R software 3.0.2 [43]. Pre-oviposition time was analysed using a Wilcoxon rank-sum test. The number of days until first oviposition (pre-oviposition time) was compared between control and infected insects in every temperature and parasite species. Data on egg hatching rate and e-values (dependent variables) were analysed to compare the effects of: a) parasite infection (i.e. control *vs* infected insects) during three consecutive feeding cycles in every parasite treatment; b) *T. cruzi* infection (i.e. control *vs* infected insects) at two experimental temperatures (i.e. 25°C *vs* 30°C); and c) infection (i.e. control *vs* infected insects) of either parasite species (i.e. *T. cruz*i and *T. rangeli*) at the same temperature (i.e. 25°C). The variable individual was set as a random effect to account for repeated measures. Hatching rate data were analysed by a binomial generalized mixed-effects model (function glmer() in “lme4” package) [44] using “logit” as a link function. E-value data were analysed with a linear mixed-effects model (function lme() in “nlme” package) [45]. Full models with an interaction term were fitted initially and they were reduced to main effects models if the interaction was not significant. The goodness-of-fit of the models was visually inspected using the residual plots. Overdispersion was also checked for all models. *Post-hoc* interaction contrasts (function testInteractions() in “phia” package, De Rosario-Martinez [46] were performed to further explore the influence of each treatment in the data.

## Results

### Effect of trypanosome infection on *R. prolixus* reproduction

#### Pre-oviposition tim

**Figure 1:**
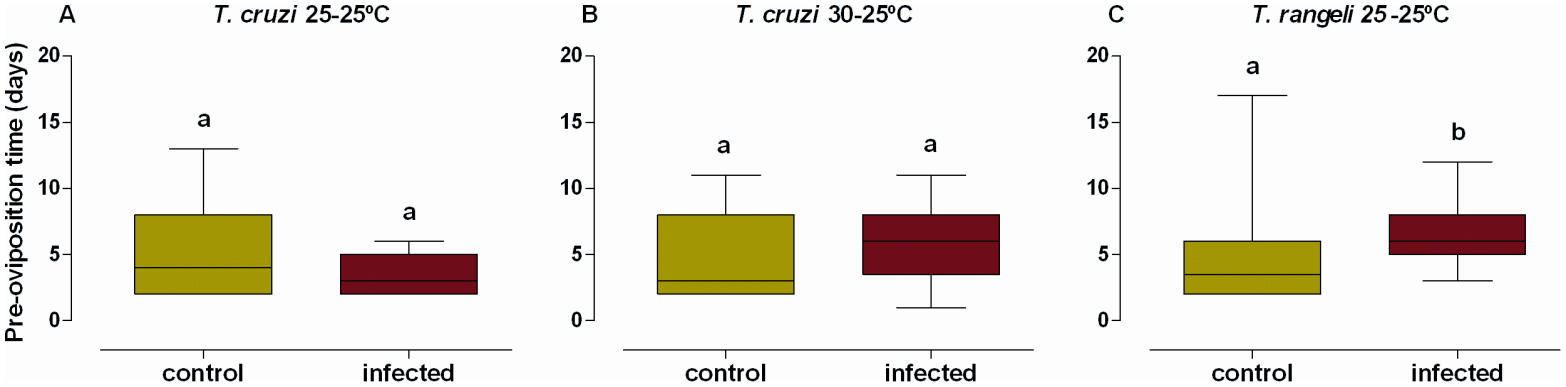
Pre-oviposition time of *R. prolixus* females infected. A) *T. cruzi* at 25–25°C; B) *T. cruzi* at 30–25°C and C) *T. rangeli* at 25–25°C. The median, quartiles and minimum and maximum numbers of days before the first oviposition are shown in each box plot. Data represent the mean ± s.e. of 15 control and 19 *T. cruzi* 25–25°C infected pairs (A), 20 control and 20 *T. cruzi* 30–25°C infected pairs (B) and 20 control and 20 *T. rangeli* 25–25°C infected pairs (C). Green indicates data from control insects, while red indicates data from infected insects. Distinct letters above columns indicate significant differences in pre-oviposition time (P<0.05).

Infection by *T. cruzi* did not affect the pre-oviposition time shown by females (Fig. 1A and 1B, Wilcoxon rank-sum test, W = 178.5, P = 0.33 and W = 152.5, P = 0.30, respectively at 25–25°C or 30–25°C). Infection of females by *T. rangeli*, however, induced a 2 day delay in their pre-oviposition time in comparison to uninfected females (Fig. 1C, Wilcoxon rank-sum test, W = 90.5, P<0.01).

#### E-value and fertility of insects infected by T. cruzi 25–25°C

**Figure 2:**
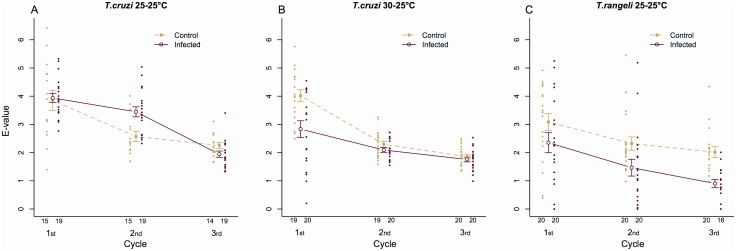
E-value of *R. prolixus* pairs infected. A) *T. cruzi* at 25–25°C; B) *T. cruzi* at 30–25°C and C) *T. rangeli* at 25–25°C. Data represent the mean e-value (± s.e.) of 15 control and 19 *T. cruzi* 25–25°C infected pairs (A), 20 control and 20 *T. cruzi* 30–25°C infected pairs (B) and 20 control and 20 *T. rangeli* 25–25°C infected pairs (C). Green indicates data from control insects, while red indicates data from infected insects. Cycles comprise periods of 21 days between meals and dots represent the e-value shown by each pair for a particular cycle.

**Figure 3:**
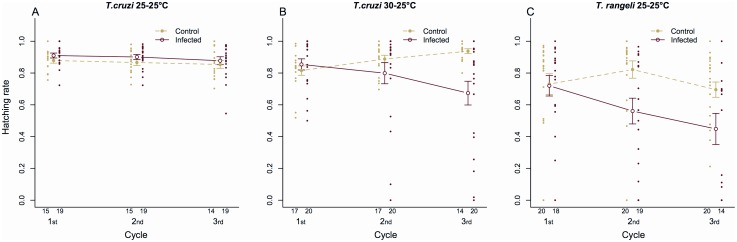
Hatching rate of *R. prolixus* eggs from pairs infected. A) *T. cruzi* at 25–25°C; B) *T. cruzi* at 30–25°C and C) *T. rangeli* at 25–25°C. Data represent the mean ± s.e. of 15 control and 19 *T. cruzi* 25–25°C infected pairs (A), 17 control and 20 *T. cruzi* 30–25°C infected pairs (B) and 20 control and 19 *T. rangeli* 25–25°C infected pairs (C). Green indicates data from control insects, while red indicates data from infected insects. Cycles comprise periods of 21 days between meals and dots represent the hatching rate shown by each pair for a particular cycle.

For *T. cruzi* 25–25°C a significant interaction between infection and cycle was observed in the e-value (Linear mixed-effects model, P = 0.01), indicating that the effect of infection depends on adult age. *Post-hoc* tests indicated a significant increase in the e-value of infected insects during the second cycle when compared to controls (Fig. 2A, control = 2.57±0.18, infected = 3.45±0.18, P = 0.004). Additionally, the hatching rate observed for infected insects was marginally increased (Fig. 3A, control = 0.87±0.01, infected = 0.90±0.01, binomial generalized mixed-effects model, P = 0.03).

#### *E-value and fertility* of insects infected by *T. cruzi* 30–25°C

The analysis of the e-value of insects exposed to *T. cruzi* infection at 30–25°C revealed a strong interaction with the cycle, indicating that infection decreased the fertility of insects at particular phases of adult life (Linear mixed-effects model, P<0.001). Infected pairs produced eggs less efficiently during the first reproductive cycle in comparison to controls (Fig. 2B, control = 4.02±0.21, infected = 2.83±0.29, P<0.001). Nevertheless, no significant differences in e-values of control and infected insects were observed in the subsequent reproductive cycles (P = 0.65 for both cycles).

An interaction between infection and cycle was also seen in the egg hatching rates (Binomial generalized mixed-effects model, P<0.001), indicating that the decrease in hatching success of eggs laid by infected pairs also depended on adult age. Results from *post-hoc* tests showed that the hatching rate of these insects decreased significantly in the third reproductive cycle as compared to that of control group insects (Fig. 3B, control = 0.94±0.01, infected = 0.67±0.07, P<0.001)

#### E-value and fertility of insects infected by T. rangeli 25–25°C

The e-value of *Trypanosoma rangeli*-infected insects was affected both by infection (Linear mixed-effects model, P = 0.001) and cycle (P<0.001), but no significant interaction between the two treatments was observed. Figure 2C shows that *T. rangeli*-infected insects suffered a slight decrease in their e-value in all cycles compared to that shown by insects of the control group. Nevertheless, this difference was statistically significant during the second (control = 2.32±0.24, infected = 1.46±0.30; P = 0.04) and third (control = 2.02±0.19, infected = 0.91±0.14, P = 0.02) cycles. Moreover, infection status and reproductive cycle showed interactive effects on hatching rates (Binomial generalized mixed-effects model, P<0.001). In fact, parental infection by *T. rangeli* significantly decreased the hatching rate of eggs laid during the second (control = 0.82±0.05, infected = 0.56±0.08, P = 0.006) and third (control = 0.70±0.05, infected = 0.45±0.10, P = 0.03) cycles (Fig. 3C).

### The effect of temperature on the outcome of *T. cruzi* infection

#### The e-value and fertility of insects infected by T. cruzi 25–25°C vs T. cruzi 30–25°C

**Figure 4:**
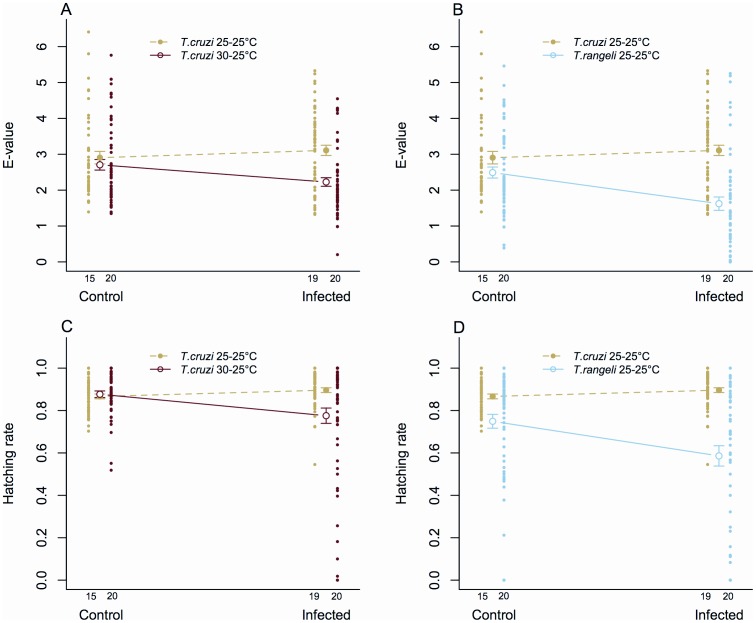
Comparison of the effect of trypanosome infection on e-value and egg hatching rate of *R. prolixus*. A) Variation of e-value between insects exposed to infection by *T. cruzi* 25–25°C and *T. cruzi* 30–25°C and their corresponding controls; B) variation of e-value between insects exposed to infection by *T. cruzi* 25–25°C and *T. rangeli* 25–25°C and their corresponding controls; C) variation of egg hatching rate between insects exposed to infection by *T. cruzi* 25–25°C and *T. cruzi* 30–25°C and their corresponding controls; D) variation of egg hatching rate between insects exposed to infection by *T. cruzi* 25–25°C and *T. rangeli* 25–25°C and their corresponding controls. Data represent the mean ± s.e. of 15 control and 19 *T. cruzi* 25–25°C infected pairs (A–D), 20 control and 20 *T. cruzi* 30–25°C infected pairs (A and C) and 20 control and 20 *T. rangeli* 25–25°C infected pairs (B and D). Green indicates data obtained from insects infected by *T. cruzi* 25–25°C and controls (A–D), red indicates data from insects infected by *T. cruzi* 30–25°C and controls (A and C) and blue indicates data from insects infected by *T. rangeli* 25–25°C and controls (B and D). Dots represent e-value and hatching rates shown by each pair.

A statistically significant interaction between infection status and experimental temperature (Linear mixed-effects model, P = 0.007) indicates that *T. cruzi* affected the efficiency of egg production significantly depending on the temperature to which insects were exposed during nymphal phases. Although there were no significant differences in the e-value of control insects exposed to either temperature treatment (P = 0.84), infected insects exposed to 30–25°C experienced significant e-value reduction compared to those kept at 25–25°C (Fig. 4A, 25–25°C = 3.11±0.14, 30–25°C = 2.23±0.12, P<0.001).

Similar results were encountered in egg-hatching rates. A significant interaction found after comparing hatching rates as a function of insect infection by *T. cruzi* and temperature (Binomial generalized mixed-effects model, P = 0.02) indicates that eggs laid by infected pairs vary their hatching success depending on the temperatures to which they were exposed. The hatching of eggs laid by infected insects exposed to 30–25°C was significantly lower when compared to that of bugs at 25–25°C (Fig. 4C, 25–25°C = 0.90±0.01, 30–25°C = 0.78±0.04, P = 0.009). A similar tendency was not observed in non-infected bugs (P = 0.56).

### The effect of different parasite infection

#### The e-value and fertility of insects infected by T. cruzi 25–25°C vs T. rangeli 25–25°C

The effects of infection on insects kept at the same temperature depended on parasite species, as reinforced by results obtained with insects infected by *T. cruzi* 25–25°C or *T. rangeli* 25–25°C (Linear mixed-effects model, P = 0.002). In fact, infection by *T. rangeli* strongly decreased the e-value of insects compared to that of bugs infected with *T. cruzi* (Fig. 4B, *T* *. cruzi* = 3.11±0.14, *T. rangeli* = 1.62±0.19, P<0.001).

The egg-hatching rates were clearly influenced by parasite species (Binomial generalized mixed-effects model, P = 0.02), as there was a hatching rate reduction on the eggs laid by *T. rangeli* infected pairs when compared to that of eggs laid *T. cruzi* infected insects (Fig. 4D, *T* *. cruzi* = 0.90±0.01, *T. rangeli* = 0.59±0.05, P<0.001).

## Discussion

We initially aimed to evaluate reproductive performance effects of *T. cruzi* infection on *R. prolixus* adults. For this we chose to expose insects to temperatures they are known to prefer, i.e. 25°C [47]. We also evaluated whether parasite load would affect this outcome by comparing these results to those obtained with a group of insects held at 30°C to promote a higher parasitemia. Our results have shown for the first time that *T. cruzi* infection can impose costs on *R. prolixus* reproduction, which costs we would expect to see reflected in the insect's overall fitness. This seems to be the outcome of an interplay between parasite infection, insect age and environmental temperature. We have also shown that *T. rangeli* infection is costly for the reproductive performance of these bugs

Curiously, while *T. rangeli* is generally considered a pathogen of triatomines [27], [31], [34], *T. cruzi* has been mostly considered non-pathogenic to its insect vectors [17], [18], [20]. Our results seem to indicate that both trypanosomes can impose costs on their invertebrate hosts, depending in part on the environmental conditions governing their interaction. Several studies have shown negative effects of parasitism on vector hosts in a other insect models: *Plasmodium* spp. *vs Anopheles* and *Aedes* mosquitoes [12], [48], Dengue virus *vs Aedes* mosquitoes [49], [50] and *Leishmania* spp. *vs* sand flies [51], [52]. In order to measure the impact of parasite infections authors have evaluated parameters such as blood feeding capacity, duration of development, adult longevity, fecundity, fertility and mating performance. Moreover, effects of *T. cruzi* infection on triatomine fitness have been studied in *Panstrongylus megistus* (Burmeister, 1835) [23] and *Triatoma brasiliensis* Neiva, 1911 [22], both reared around 30°C. In the first study, infected *P. megistus* presented a significant decrease both in their fecundity and fertility [23]. Conversely, infected *T. brasiliensis* showed no significant effects on these parameters [22]. While the first insect species normally prefers temperatures lower than those at which infection effects on adult fitness were evaluated, *T. brasiliensis* prefer temperatures around 30°C and might be better adapted to deal with trypanosome infection at this temperature. Interestingly, the population growth rate of *T. cruzi* culture epimastigotes is affected by ambient temperature [unpublished data], being twice as fast at 30°C than at 27°C. Based on this, these parasites may achieve massive populations at higher temperatures if their access to nutrients is not restricted in the vector gut. In such a scenario, insects would probably lose a large amount of nutritional resources to the parasite population. Moreover, immunological responses to control parasite development would impose an additional energetic cost [10], [53]. Comparison of our results and those of previous reports seem to suggest that the environmental temperature at which insects were reared, as well as their adult age, have an impact in the outcome of triatomine-trypanosome interactions.

We have shown that infection by *T. cruzi* does not affect the time taken to initiate oviposition by *R. prolixus* pairs. This seems to be in agreement with a previous study [22] using *T. brasiliensis*. In our experiments, the temperature at which infected *R. prolixus* bugs were held did not have an impact on the pre-oviposition time shown as adults. Nevertheless, infection by *T. rangeli* significantly delayed the onset of oviposition by *R. prolixus* females, showing a cost in terms of reproductive capacity.

An unexpected result was the increase in the e-value of *T. cruzi*-infected insects held at 25°C. This effect was restricted to the second feeding cycle and for these insects, hatching rates were not affected by insect age, but were increased in the infected insects. At present, we can only speculate on the causes of this phenomenon but it might be an interesting topic to investigate further, especially given the possibility of environment-dependent costs of infection and even that costs become benefits under some situations. The phenomenon demonstrated here on *T. cruzi*-infected insects at 25°C has a profile similar to the hormesis reaction, characterized by a reversal of response between low and high doses of a stressor [see review 54].

The negative effects observed on *T. cruzi*-infected insects reared at 30°C may be the consequence of a higher parasite load affecting adult bug energetic balance. In fact, insects exposed to this treatment presented significant decreases in their e-value and the hatching rates of their eggs. The fact that the decrease in e-value was observed during the first reproductive cycle reinforces the idea of negative effects being triggered due to large parasite populations competing for nutrients with bugs. Insects were transferred to 25°C after imaginal molt to avoid inducing a reduction in their nutritional resources due to an increased metabolic rate at higher temperatures. In this way, the probably large parasite populations they bore might have impacted their nutritional resources, promoting the negative effects observed on e-value. In the next cycles, a combination of lower temperature (slowing parasite growth) and cyclic starvation (decreasing available resources) might have decreased parasite populations bringing them under a threshold where reproductive performance is not affected. However, even if *T. cruzi*-infected insects managed to maintain fecundity parameters similar to those of uninfected insects during the last two cycles studied, the quality of their eggs was significantly affected by infection as shown by their diminished hatching rates. Moreover, this reproductive parameter was affected by insect age, as the proportion of viable offspring decreased as pairs became older. This decreased reproductive performance of *T. cruzi*-infected insects exposed to higher temperatures might be a consequence of direct competition for energetic resources that could otherwise be used for reproduction [55], an outcome of committing extra resources to implementing immune responses [56] or a result of host manipulation by parasites that reduce host investments in fecundity and prolong parasite survival [55]. To our knowledge, this is the first time that the e-value was used to evaluate the impact of trypanosome infection on triatomines.

Infection by *T. rangeli* affected all reproductive parameters evaluated in the present study. As mentioned above, infected pairs showed delayed pre-oviposition times and both their e-values and hatching rates were significantly decreased. Moreover, these negative effects increased with insect age. The virulence of *T. rangeli* to triatomines has been broadly described and shown to vary according to parasite strain and experimental methodologies [57]. However, effects of *T. rangeli* on the reproductive performance of triatomines have not been shown to date. In this sense, our results show an additional negative effect that *T. rangeli* can cause to its insect vector. Differently from *T. cruzi,* that develops exclusively in the intestinal tract of bugs, parasites of this species can cross the intestinal epithelium and continue their development in the hemocoel and salivary glands. As the eggs of these insects need abundant energy reserves, females undergo an intense mobilization of lipids, proteins and carbohydrates during oogenesis [58], [59]. In *R. prolixus* females, lipids are transported from the fat body to the ovaries by a lipoprotein called lipophorin, to allow their incorporation by oocytes [60], [61]. As trypanosomes lack the complete synthesis pathways for some fundamental lipids they must obtain these from their hosts [62]–[65]. *Trypanosoma rangeli* epimastigotes incorporate lipids together with lipophorin from the host hemolymph [66], probably decreasing the amount of these protein carriers available to the host. In addition, infection by *T. rangeli* can result in large parasite populations found in both the intestine and hemolymph [9]. This could also decrease insect nutritional resources and impact fecundity and fertility. It is important to highlight that pairs were allowed to copulate *ad libitum* in all treatments, even though we did not control whether uninfected insects copulated more frequently than infected ones. It is known that a greater mating frequency improves the fecundity shown by triatomine pairs [67], [68] and the possibility of consequences of trypanosome infection on bug mating performance deserves to be analyzed in further studies.

## Conclusions

The reproductive performance of *R. prolixus*, a key vector for Chagas disease transmission to humans, has been shown here to diminish due to trypanosome infection. This can reduce the insect's fitness and therefore exert selective pressures on their interaction with the parasites. It is still not clear whether infection induced a decrease in the reproductive performance of males, females or both, since we have not performed assays to check this possibility, i.e., tests with pairs of infected females mating with control males or infected males with control females. We suggest that much of the selective pressures may be manifested in behavioral effects – infected insects may select a thermal environment that ameliorates negative effects of the parasites, for example, and it is quite possible that mate choice is affected by infection status. It would also be interesting to investigate the fitness of the offspring of infected parents and other subtle, yet ecologically (and therefore epidemiologically) relevant, aspects of these vector-parasite interactions. As mentioned above, we have recently determined that trypanosome-triatomine interactions are drastically affected by environmental temperature, showing that parasite pathogenicity is modulated by this parameter (unpublished data). This might suggest that other known stressors such as starvation and low water vapor pressure may expose insects to conditions inducing significant pathogenicity by these parasites.
